# Comparative genomic and phenomic analysis of *Clostridium difficile* and *Clostridium sordellii*, two related pathogens with differing host tissue preference

**DOI:** 10.1186/s12864-015-1663-5

**Published:** 2015-06-10

**Authors:** Joy Scaria, Haruo Suzuki, Christopher P. Ptak, Jenn-Wei Chen, Yongzhang Zhu, Xiao-Kui Guo, Yung-Fu Chang

**Affiliations:** Department of Population Medicine and Diagnostic Sciences, College of Veterinary Medicine, Cornell University, Ithaca, NY 14853 USA; Department of Veterinary and Biomedical Sciences, South Dakota State University, Brookings, SD 57007 USA; Graduate School of Science and Engineering, Yamaguchi University, Yamaguchi, Japan; Department of Medical Microbiology and Parasitology, Institutes of Medical Sciences, Shanghai Jiao Tong University School of Medicine, Shanghai, 200025 China

**Keywords:** Clostridium difficile, Clostridium sordellii, Comparative genomics, Phenotype microarray, Urease

## Abstract

**Background:**

*Clostridium difficile* and *C. sordellii* are two anaerobic, spore forming, gram positive pathogens with a broad host range and the ability to cause lethal infections. Despite strong similarities between the two Clostridial strains, differences in their host tissue preference place *C. difficile* infections in the gastrointestinal tract and *C. sordellii* infections in soft tissues.

**Results:**

In this study, to improve our understanding of *C. sordellii* and *C. difficile* virulence and pathogenesis*,* we have performed a comparative genomic and phenomic analysis of the two. The global phenomes of *C. difficile* and *C. sordellii* were compared using Biolog Phenotype microarrays. When compared to *C. difficile*, *C. sordellii* was found to better utilize more complex sources of carbon and nitrogen, including peptides. Phenotype microarray comparison also revealed that *C. sordellii* was better able to grow in acidic pH conditions. Using next generation sequencing technology, we determined the draft genome of *C. sordellii* strain 8483 and performed comparative genome analysis with *C. difficile* and other Clostridial genomes. Comparative genome analysis revealed the presence of several enzymes, including the urease gene cluster, specific to the *C. sordellii* genome that confer the ability of expanded peptide utilization and survival in acidic pH.

**Conclusions:**

The identified phenotypes of *C. sordellii* might be important in causing wound and vaginal infections respectively. Proteins involved in the metabolic differences between *C. sordellii* and *C. difficile* should be targets for further studies aimed at understanding *C. difficile* and *C. sordellii* infection site specificity and pathogenesis.

**Electronic supplementary material:**

The online version of this article (doi:10.1186/s12864-015-1663-5) contains supplementary material, which is available to authorized users.

## Background

The bacterial class, *Clostridia*, is typified by gram-positive anaerobes and includes several important human pathogens. The main virulence factors produced by pathogenic *Clostridia* are secreted toxins. While *C. botulinum* (botulism) and *C. tetani* (tetanus) are the best known of these pathogens, other members, in particular *C. difficile*, have become increasingly notorious due to an accelerating number of documented infections in recent years. In North America and Europe, *C. difficile* infection (CDI) is now the leading cause of infectious diarrhea [1–3]. CDI can cause a varying range of diseases from mild diarrhea to fulminant colitis and death [4–6]. The primary risk factors of CDI include antibiotic treatment, advanced age, severe underlying illness, prior hospitalization, tube feeding, gastrointestinal surgery, and use of proton-pump inhibitors [7, 8]. *C. difficile* also has a broad host range and causes infection in agriculturally important animals such as pigs, cattle, horses and chickens [9–13].

Recent studies on the *Clostridium* genus support a reclassification of *C. difficile* and the related Cluster XI into a family-level group that is distinct from the current *Clostridiaceae* family (renaming family genus spp. to *Peptostreptococcaceae Peptoclostridium difficile* has been suggested) [14]. Along with *C. difficile*, Cluster XI currently includes several clinically significant members, *C. sordellii*, *Filifactor alocis*, and *Peptostreptococcus anaerobius* [15–17]. *C. sordellii* infection (CSI), although not as prevalent as CDI, has a very high mortality rate that can often reach 75 % lethality [15]. Both *C. sordellii* and *C. difficile* are asymptomatically carried in the gastrointestinal tracts of about 10 % of adult humans [15, 18] and both species also can infect animals [19–22]. In addition, *C. sordellii* and *C. difficile* excrete potent toxins with immunological cross-reactivity and similar biological activities [15, 23, 24]. Despite the close similarities in host range and virulence factors, there are two striking differences between *C. difficile* and *C. sordellii.* First is that while *C. difficile* only colonizes the gastrointestinal tract, *C. sordellii* can colonize both the human gastrointestinal tract and vagina [25]. Secondly, while *C. difficile* infection affects the host intestine, *C. sordellii* primarily causes soft tissue infection. The tissue preference of *C. sordellii* results in CSI being primarily reported among reproductive-age women following natural childbirth, spontaneous, surgical or medical abortions [15, 26]. Wounds from illicit injectable drug use, non-gynecological surgical procedures, penetrating crushing injuries, or traumatic injury in previously healthy men, women, and children can also lead to CSI infection [27–32].

Comparative analyses of closely related bacteria with different infection site specificity and pathogenicity can provide information relevant to understanding adaptation to host environments and mechanisms of infection. Genomic differences can lead to phenotype level changes. In bacteria, phenotypic variations are often related to metabolic changes, which are defined by the ability to utilize various sources of carbon, nitrogen, sulfur, phosphorous, and other essential nutrients. With the development of Phenotype microarrays (PMs), high-throughput determination of a microorganism’s global metabolic phenotype or phenome is now possible [33–37]. In this study, to determine the genomic and phenomic basis for the differences in *C. sordellii* and *C. difficile* infections, we have performed a comparative genomic and phenomic analysis of these two species. The global phenome of *C. sordellii* and *C. difficile* revealed several differences, most notably in acid resistant growth. We further explored the genomic basis for phenomic differences by determining a draft genome sequence of *C. sordellii* strain 8483 and comparing it against two *C. sordellii* genomes (strains ATCC_9714, and VPI_9048) and eight *C. difficile* genomes (strains 630, BI1, CD196, M68, R20291, 2007855, CF5, and M120). Understanding the differential adaptions to host tissue at the genomic and phenomic level should provide opportunities in the fight against these important infections.

## Results and discussion

### Nutritional phenomic comparison

In this study, we have performed the comparative analysis of the genome and phenome of the two closely related Clostridial pathogens, *C. sordellii* and *C. difficile*. To determine the global phenome of *C. sordellii* and *C. difficile*, we used Biolog phenotype microarrays (PMs) which enable whole cellular level determination of bacterial phenotypes [35]. The nutritional PM analysis consisted of 190 assays of carbon source metabolism, 94 assays of phosphorous and sulfur source metabolism, 95 assays of biosynthetic pathways, and 380 assays of nitrogen source metabolism [35]. We have previously analyzed the phenome of six *C. difficile* strains [38]. We compared those results with phenome of *C. sordellii* strain 8483 determined in this study. From a total of 759 nutritional phenotype assays, 160 were positive (indicated by a 40 % growth enhancement relative to the control) for *C. sordellii* strain 8483, while 132 were positive for *C. difficile* strain 630 (Additional file 1: Table S1 and Additional file 2: Table S2). *C. sordellii* and *C. difficile* shared 65 positive phenotypes while 162 positive phenotypes were specific to just one of the two species. A disproportionate number of unshared phenotypes were identified in the carbon and nitrogen source assays (~3.0× unshared vs. shared for carbon and nitrogen combined; ~1.3× unshared vs. shared for other sources combined). The unshared carbon and nitrogen phenotypes are indicative of species specific adaptations to environmentally available nutrient sources.

**Fig. 1:**
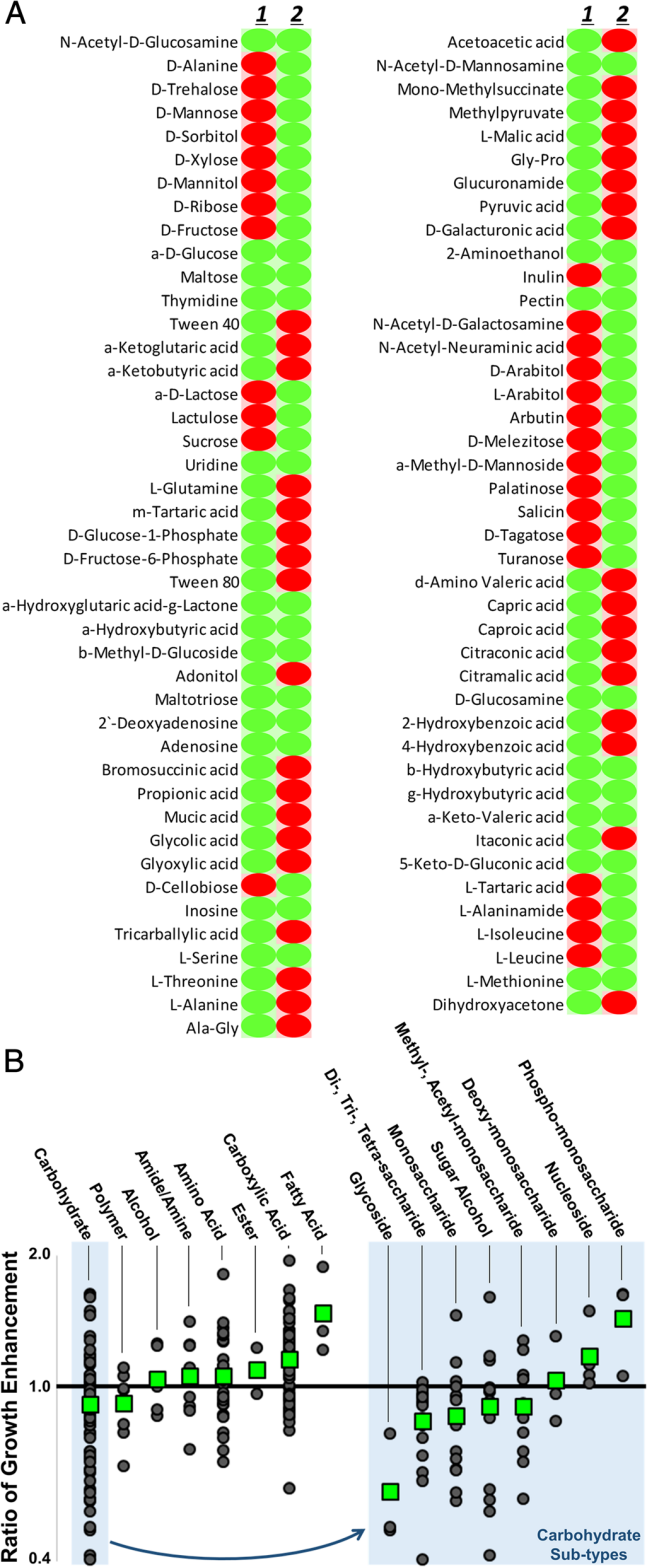
Carbon source utilization microarray. **a** The list includes all positive carbon source phenotypes (green) for *C. sordellii* and *C. difficile* (lanes 1 and 2, respectively). Negative phenotypes are red. **b** The *C. sordellii* : *C. difficile* ratio of growth enhancement for each carbon source phenotype (gray circle) is grouped by carbon source type. The average for each group is indicated (green box). The carbohydrate group was further divided into sub-types

A basic illustration of the carbon source preference exhibited by each species is depicted in a table listing all 85 positive carbon phenotype conditions (Fig. 1a). A *C. sordellii* to *C. difficile* ratio of growth enhancement was used to more directly compare the ability of the two species to utilize specific carbon sources (Fig. 1b). The tested carbon source phenotypes were grouped based on the type of carbon source molecules. *C. difficile* more effectively utilized typical carbohydrates, while *C. sordellii* was better able to utilize non-carbohydrates, including amino acids and fatty acids, as carbon sources. Carbon sources grouped as carbohydrates were further divided into molecular sub-types resulting in a similar trend. Most typical saccharides could be utilized more effectively by *C. difficile* than by *C. sordellii*, yet saccharides that have been modified for incorporation into nucleic acids or phosphorylated for entering in metabolic pathways can be more easily utilized by *C. sordellii*. The difference in ability to exploit a broad range of non-carbohydrate molecules as carbon sources could improve the likelihood of *C. sordellii* to survive within the more complex environment of soft tissue.

**Fig. 2:**
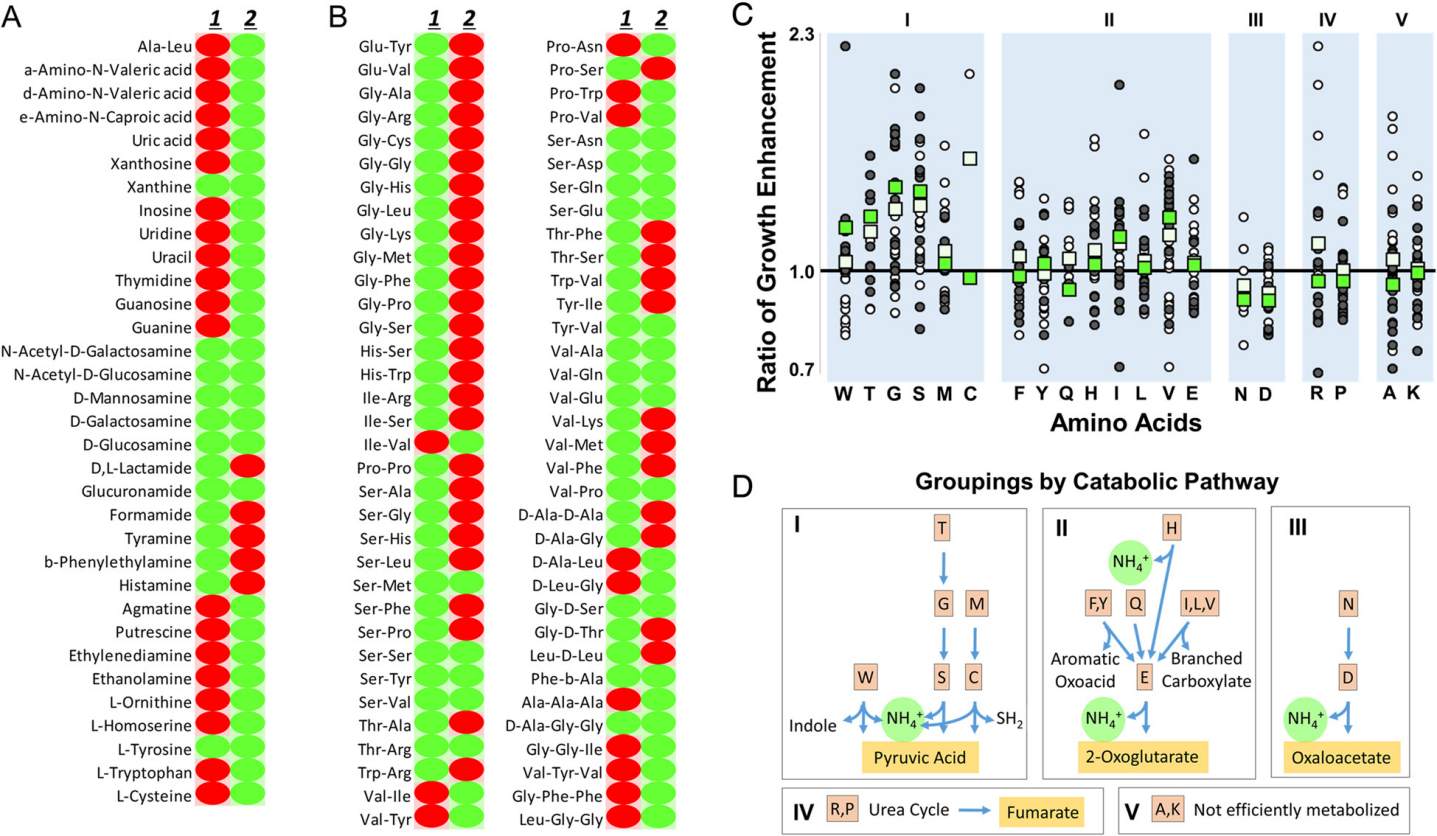
Nitrogen source utilization microarray. The lists include all positive simple (**a**) and di- and tri-peptide (**b**) nitrogen source phenotypes (green) for *C. sordellii* and *C. difficile* (lanes 1 and 2, respectively). Negative phenotypes are red (**c**). The *C. sordellii* : *C. difficile* ratio of growth enhancement for each dipeptide nitrogen source phenotype is grouped by amino acid (circles; average – white squares). Gray circles indicate the position 1 amino acid (position 1 average – green squares) while white circles indicate the position 2 amino acid. Amino acids are further grouped (I-V) by their expected pathway to nitrogen metabolism (**d**)

Figures 2a and 2b list all 101 positive nitrogen and peptide nitrogen source phenotype conditions, respectively. The positive phenotype threshold for nitrogen source growth enhancement was achieved for more than 2× as many simple nitrogen source conditions by *C. difficile* than by *C. sordellii*; however, *C. sordellii* was able to reach the positive phenotype threshold for nearly 2× as many peptide nitrogen conditions as *C. difficile*. For example, *C. difficile* could better utilize the simple amino alcohol, ethanolamine, which is an abundant nitrogen source in the gut. As *C. sordellii* was better able to utilize protein building blocks as carbon sources, the fact that this preference extends to nitrogen sources is not surprising. A *C. sordellii* to *C. difficile* ratio of growth enhancement was used to compare nitrogen source utilization between the two species for the 246 di-peptide nitrogen source phenotypes. The ratios were grouped by the presence of amino acids in the di-peptide sources and organized into higher level groupings by their expected catabolic pathway leading to nitrogen metabolism as defined by available KEGG pathway enzymes [39] in the *C. sordellii* genome (Fig. 2c). Because the di-peptide nitrogen source microarray was not comprehensive, the amino acid sample size was variable. For most amino acids, data was obtained from between 22 and 36 di-peptide conditions. Ratios for asparagine (n = 9), glutamine (n = 14), and threonine (n = 15) trended similarly to amino acids within the same grouping. The sample size for cysteine di-peptides (n = 2) was small and only incorporated glycine, but interestingly the growth ratio was strongly dependent on amino acid position. Analysis of only the first amino acid was also included in Fig. 2c since the distributions were generally narrower than when both amino acid positions 1 and 2 were included. Amino acids in group I are likely to follow a pathway that generates pyruvic acid when ammonia is liberated as a nitrogen source (Fig. 2d). The largest bias for *C. sordellii* growth enhancement was observed for group I amino acids: glycine, serine, and threonine which are linked to the same pathway. The other notable amino acid group (III) contains aspartic acid and asparagine, which are catabolized to oxaloacetate, and are more effectively utilized by *C. difficile* than by *C. sordellii*. In comparison to *C. difficile*, *C. sordellii* has adapted to the broader use of available peptides (glycine, serine, and threonine are of relatively high abundance in human genes [40]) as nutrient sources, which could help *C. sordellii* infiltrate soft tissue.

Our analysis finds that, at the phenome level, both of these closely related species share a core group of functions and phenotypes. Our phenotype array results also reveal some key phenotype differences particularly for carbon and nitrogen source utilization that might explain the differences in the primary site of infections caused by these two species. Both glycolysis and amino acid catabolism are differentially regulated in *C. difficile* suggesting a possible mechanism for how these pathways have evolved to better occupy the respective niche for both *C. sordellii* and *C. difficile* [41].

### Osmolyte and pH phenomic comparison

**Fig. 3:**
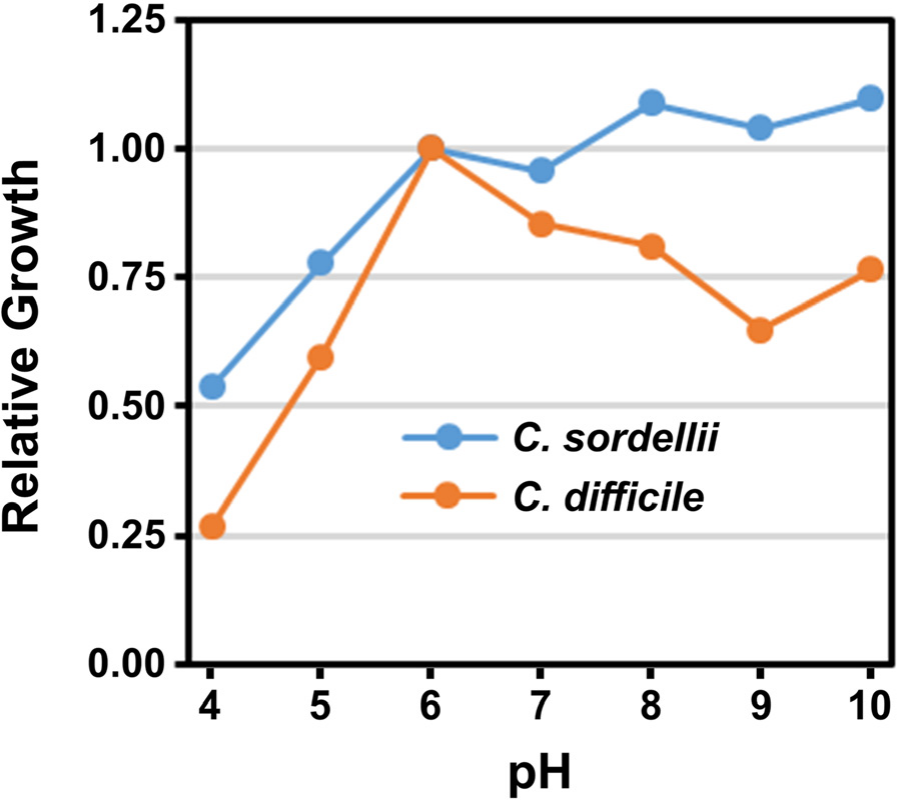
Growth dependence on pH. Phenotype Microarray growth data for the pH range 4–10 was obtained from PM10-A (Additional file 1: Table S1). For each species, the data was normalized to pH 6, which is the optimal growth pH of *C. difficile*

Two additional PM arrays were utilized for osmolyte and pH sensitivity (Additional file 1: Table S1). Optimum growth was obtained at pH 6.0 for *C. difficile*. An analysis of pH-dependent growth normalized to growth at pH 6.0 for each species revealed that *C. sordellii* also achieved near-optimal growth levels at pH 6.0 (Fig. 3). Further, *C. sordellii* performed better than *C. difficile* at off-optimal growth pHs. At pH 4.0, growth of *C. sordellii* is 2× as robust as *C. difficile* relative to growth at pH 6.0. Because the normal, healthy pH of the vagina is pH 4.5 or lower, the ability of *C. sordellii* to grow at acidic pH plays a crucial role in facilitating vaginal infections [42]. Fecal contamination of the vagina during vaginal delivery could provide a source of organisms that may infect vaginal tears, episiotomy sites or ascend to the uterus through the open cervix [15]. Interestingly, *C. sordellii* was able to maintain similar growth levels from pH 6 through pH 10 while the growth of *C. difficile* was modestly reduced above pH 7 suggesting that *C. sordellii* has an improved survivability at both acidic and basic pHs.

*C. difficile* showed better adaptation to growth under 78 % of osmolyte conditions; however, *C. sordellii* had a growth advantage over *C. difficile* in 83 % of urea-specific conditions. The combination of low pH and urea led to a > 40 % growth enhancement for *C. sordellii* over pH 6.0 conditions while the same combination inhibited growth in *C. difficile* by > 40 % relative to optimal growth at pH 6.0. *C. sordellii* has previously been shown to exhibit urease activity [43]. Details on the *C. sordellii* urease gene cluster and its implications for growth improvement under multiple conditions including acidic pH are discussed in the later sections.

### Phylogeny

**Fig. 4:**
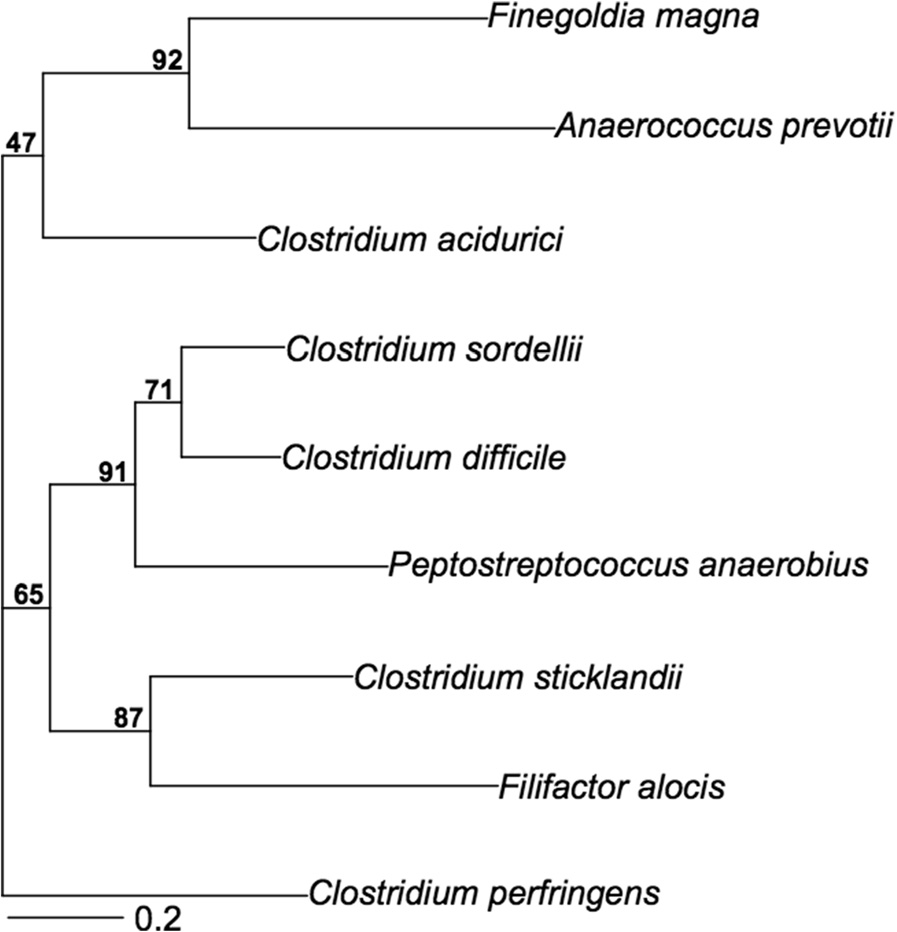
Phylogeny. Maximum likelihood tree obtained from a concatenated nucleotide sequence alignment of the 134 orthologous genes of the 9 bacterial species from Clostridial clusters I, XI, XII, and XIII. The horizontal bar at the base of the figure represents 0.2 substitutions per nucleotide site. The percentages of genes that support the branches of the tree are indicated

*Clostridium* species have been classified into different phylogenetic groups with *C. sordellii* and *C. difficile* belonging to Clostridial cluster XI [44]. In this study, 134 orthologous genes with greater than 90 % bootstrap support were identified and used to infer phylogenetic relationships of 9 bacterial species from Clostridial clusters I, XI, XII, and XIII. The phylogenetic tree inferred from a concatenation of the 134 genes (Fig. 4) agreed with and further confirmed the ribosomal proteins-based phylogeny reported in a previous study [14], placing *Clostridium acidurici* (cluster XII) as a sister group of *Anaerococcus prevotii* and *Finegoldia magna* (cluster XIII), joined by the cluster XI (*C. sordellii*, *C. difficile*, *Peptostreptococcus anaerobius*, *Clostridium sticklandii*, and *Filifactor alocis*), and finally *Clostridium perfringens* (cluster I) as an outgroup. The concatenation phylogeny demonstrated that the majority (71 %) of genes supported the monophyly of the clade of *C. sordellii* and *C. difficile*, suggesting that *C. sordellii* is a sister group to *C. difficile*.

### Gene repertoire

Species with similar gene contents should have similar functional potential as a whole [45]. Whole gene contents of any genome can be the result of a mixture of different evolutionary events such as vertical inheritance of genes and their duplication, gain and loss events, and their relative contributions may vary among different species [46]. The 22,612 proteins from the 9 bacterial species from Clostridial clusters I, XI, XII, and XIII were classified into 5979 homologous groups or protein families. Of the 5979 protein families, 3578 were present in a single strain, 2401 were present in two or more strains, of which 487 were shared by all the strains.

**Fig. 5:**
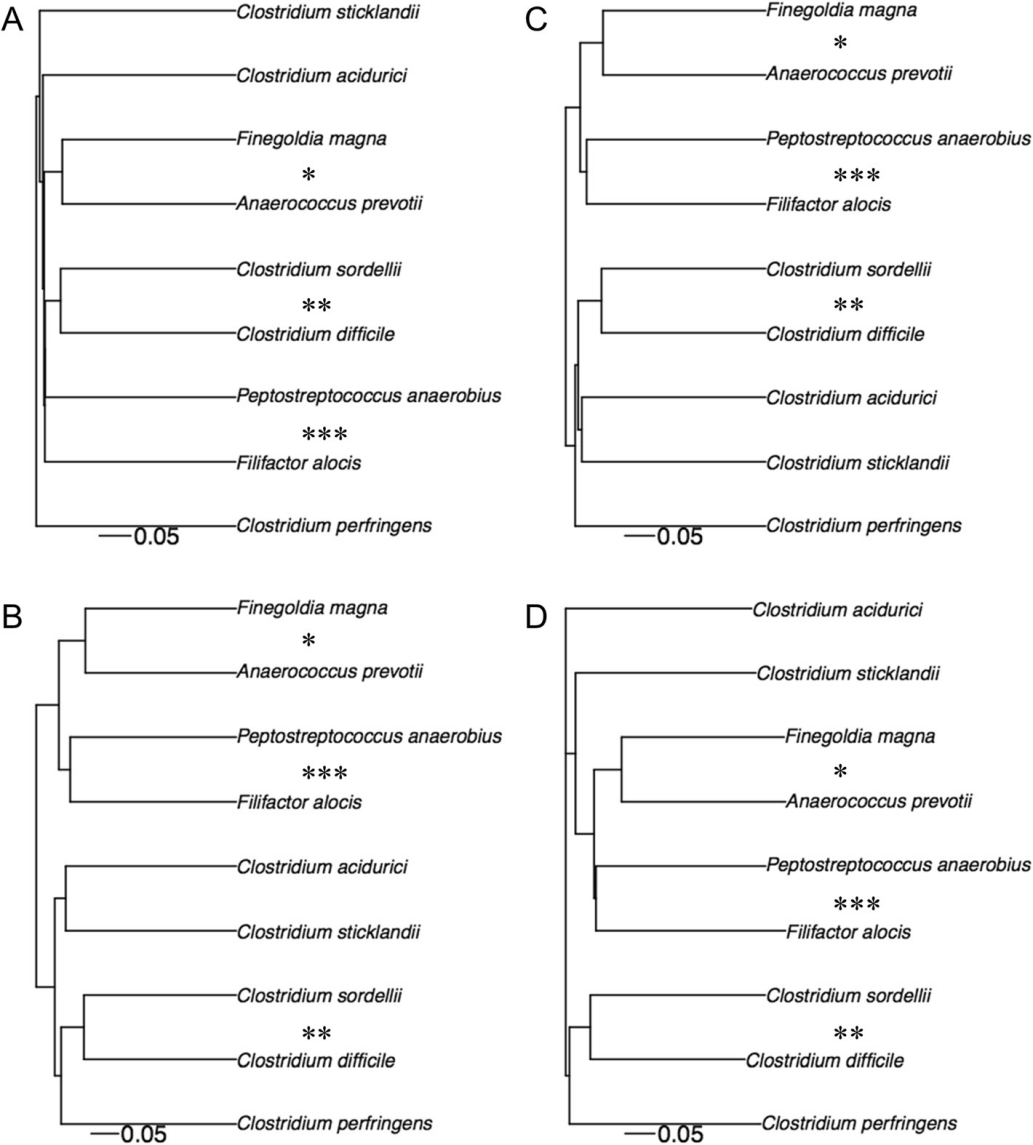
Whole gene content. Trees constructed by cluster analysis of the 9 Clostridial species based on whole gene content using four agglomeration methods: single-linkage **a**, complete-linkage **b**, average-linkage **c**, and neighbour-joining **d** clustering. Clusters formed by all four methods are identified

Cluster analysis of the 9 genomes based on the whole gene content using four agglomeration methods (i.e., single-linkage, complete-linkage, average-linkage, and neighbour-joining clustering) produced four distinct trees (Fig. 5), which were all incongruent with the concatenation phylogeny (Fig. 4). Previous studies also reported that whole gene contents of bacteria do not strictly follow their phylogenetic relationships [47–50] and can lead to a distorted ancestral lineage [51]. Although all four gene-content trees were topologically distinct and none matched the topology of the concatenation phylogeny, *C. sordellii* and *C. difficile* ubiquitously formed a single cluster (designated with ** in Fig. 5). Because all four gene-content trees suggest that *C. sordellii* and *C. difficile* are more similar to one another than to any other species, a comparison of the genomic differences might provide useful information regarding their important clinical differences.

**Fig. 6:**
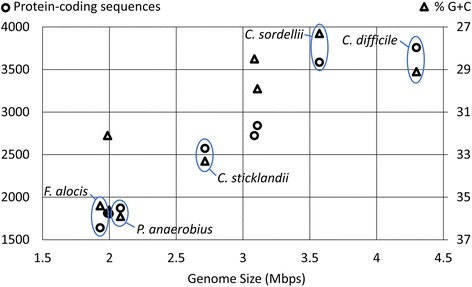
Clostridial genome size. For the 9 Clostridial species analyzed, the number of protein-coding sequences (o) are correlated with genome size (Mbps). The percentage of G + C (∆) is negatively correlated with genome size and is plotted on an inverted axis. The 5 members of Clostridial cluster XI are identified

A total of 135 protein families were present in *C. sordellii* and *C. difficile* but absent in the other 7 species. The protein families included stage III sporulation protein AF, flagellar protein FliZ, and K^+^-transporting ATPase subunits A (KdpA) and C (KdpC). A total of 296 protein families were present in *C. sordellii* and *C. difficile* but absent in the other 3 species (*P. anaerobius*, *C. sticklandii*, and *F. alocis*) in the cluster XI. Within the cluster XI, genome size and the number of protein-coding sequences (CDS) were larger in *C. sordellii* (3.6 Mbps and 3586 CDS, respectively) and *C. difficile* (4.3 Mbps and 3758 CDS, respectively) than in the 3 other species (1.9 - 2.7 Mbps and 1871–2573 CDS, respectively), while genome G + C content of *C. sordellii* (27 %) and *C. difficile* (29 %) were lower than those of the other 3 species (33 - 36 %) (Fig. 6; Additional file 3: Table S3). Our results suggest that *C. sordellii* and *C. difficile* increased their genome size and/or the other cluster XI lineages (*P. anaerobius*, *C. sticklandii*, and *F. alocis*) decreased their genome size after divergence from the common ancestor of cluster XI. The larger genome size of *C. difficile* and *C. sordellii* relative to other members of cluster XI may be linked to their unique set of shared genes.

### Gene repertoire comparison between *C. sordellii* and *C. difficile*

We compared the gene repertoire between eight *C. difficile* strains (630, BI1, CD196, M68, R20291, 2007855, CF5, and M120) and three *C. sordellii* strains (8483, ATCC_9714, and VPI_9048). Proteins from the 11 strains were classified into 4368 homologous groups (protein families); (see Additional file 4: Table S4 for a comprehensive list). Of the 4368 protein families, 928 were present in a single strain, 3440 were present in two or more strains, of which 1395 were shared by all the strains.

**Fig. 7:**
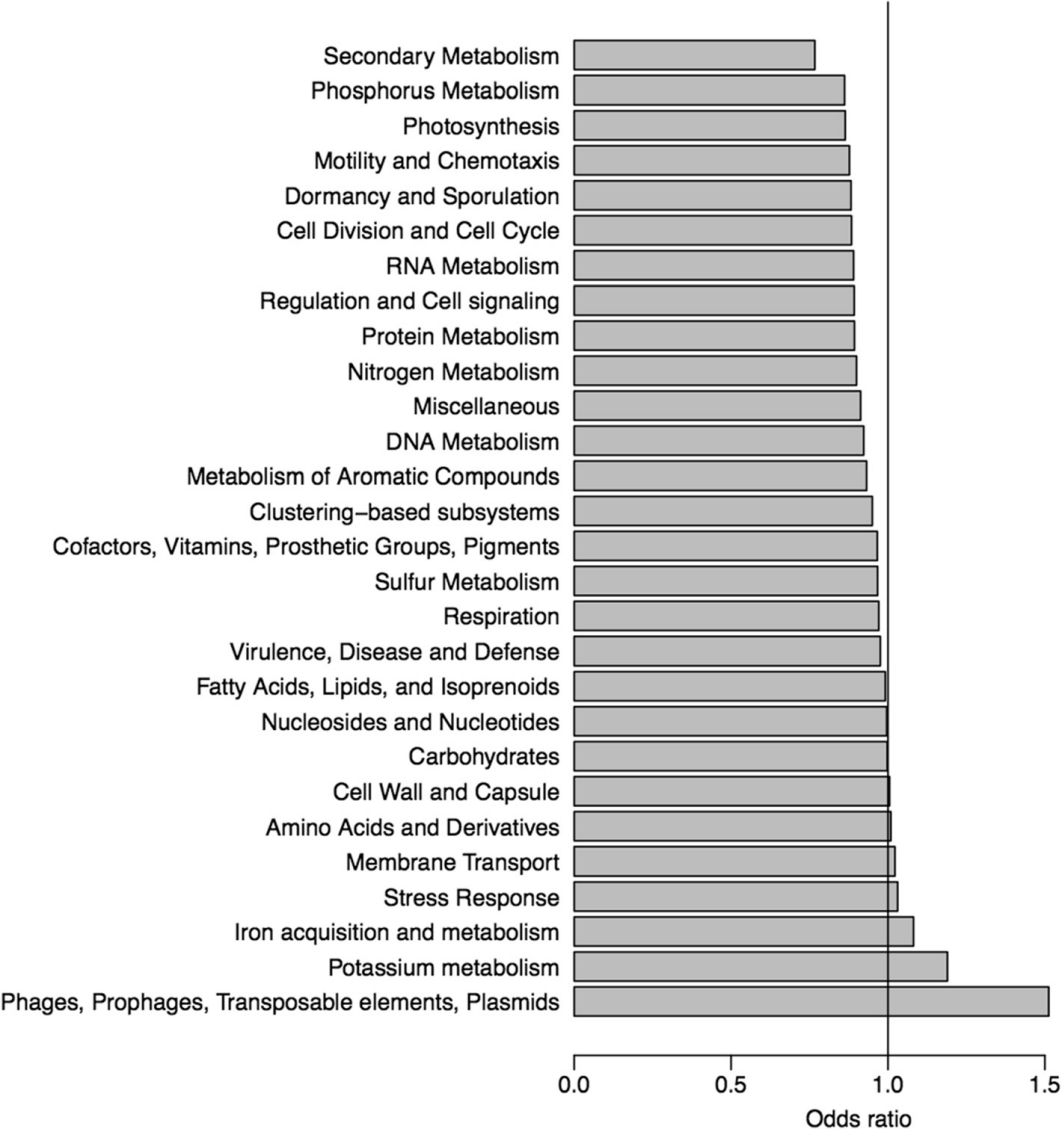
Gene set enrichment. Odds ratio to rank the relative overrepresentation (>1) or underrepresentation (<1) of each SEED category in the *C. sordellii* genome relative to the *C. difficile* genome

We performed gene set enrichment analysis to examine over- and underrepresented functional categories in *C. sordellii* relative to *C. difficile* (Additional file 5: Table S5). We calculated the odds ratio (OR) to rank the relative overrepresentation (OR > 1) and underrepresentation (OR < 1) of each functional category, and P-value of Fisher’s exact test. Fig. 7 shows the OR values of SEED subsystems as examples. The SEED subsystem “Urea_decomposition” (OD = 11.9), the Gene Ontology (GO) term “nickel cation binding (GO:0016151)” (OD = Infinite), and the Virulence Factors Database (VFDB) keywords “Acid resistance”, “Colonization”, and “Enzyme” (OD = Infinite) were significantly overrepresented in *C. sordellii* relative to *C. difficile* based on Fisher’s exact test after false discovery rate correction for multiple comparisons (FDR < 0.05).

A total of 738 protein families were present in all the 3 *C. sordellii* strains but absent in all the 8 *C. difficile* strains (Additional file 4: Table S4). These *C. sordellii*-specific genes may have been gained on the branch leading to the *C. sordellii* strain, and could be linked to its specific environmental adaptation and pathogenesis. They included some genes in the pathogenicity locus; i.e., locus_tag WS9_01807 to WS9_01812 in strain 8483, H476_0268 to H476_0289 in strain VPI_9048, and H477_0262 to H477_0286 for strain ATCC_9714 [52]. They included several amino acid decarboxylases and deaminases (e.g., glutamate decarboxylase [EC:4.1.1.15], histidine decarboxylase [EC:4.1.1.22], and L-serine deaminase) that might enable *C. sordellii* to grow on peptide nutrient sources in soft tissue. In addition, glutamate decarboxylase and arginine deiminase [EC 3.5.3.6] produce alkaline byproducts that have been suggested to participate in the acid resistance of gram-positive bacteria [53] and improve *C. sordellii* growth in acidic environments. Two protein families annotated as “KUP system potassium uptake protein (K03549)” and “potassium voltage-gated channel Shab-related subfamily B member 1 (K04885)” were present in *C. sordellii* strains but absent in *C. difficile* strains. Proteins involved in potassium transport often play a role in adaptive pH tolerance [54] and may increase broad pH range survival in *C. sordellii*.

#### SEED subsystems

The SEED annotation engine defines genes associated with a functional role in a bacterial genome as a subsystem [55]. A SEED subsystem is termed as a generalization of the term “pathway” and is a convenient framework for functional comparisons of bacterial genomes [55]. Of the 4368 protein families, 854 were assigned to the SEED subsystems (Additional file 4: Table S4). Both *C. sordellii* and *C. difficile* contained some of the important functions relevant to strain transmission and colonization. For example, both species contained 20 protein families assigned to “Dormancy and Sporulation” including sporulation sigma factor. Genes related to spore coat (cotA, cotB, cotCB, cotD, and cotE) are present in both *C. difficile* and *C. sordellii* genomes. This is consistent with the previous reports on *C. difficile* and *C. sordellii* spore properties [25, 56, 57]. Of the 14 protein families assigned to “Iron acquisition and metabolism”, hemerythrin-like iron-binding protein was present in *C. sordellii* but absent in *C. difficile*. Iron acquisition is essential for growth of pathogenic bacteria during soft tissue infections [58] and is likely to be important for *C. sordellii* in proliferating in host tissues. It has been shown that Stickland metabolism is important in *C. difficile* physiology [59]. In *C. difficile*, several genes located in D-proline reductase operon and glycine reductase operon are involved in Stickland associated metabolism. When *C. sordellii* genomes were compared to *C. difficile* genomes, most genes in the prd (prdC, prdR, prdA, prdB, prdD, prdE) and grd (grdA, grdB, grdC, grdD, grdE, and grdX) operons were found to be conserved.

#### Virulence Factors Database (VFDB)

We used the Virulence Factors Database (VFDB) [60] to assess the presence of virulence genes in the *C. sordellii* and *C. difficile* strains (Additional file 4: Table S4). Toxins A (*tcdA*) and B (*tcdB*) were homologous, and the homologous proteins were present in all the 11 genomes of *C. difficile* and *C. sordellii*. For strain ATCC_9714, locus_tag H477_0265 (Truncated TcsH) is annotated as toxin A, and H477_0263 (Cytotoxin L) is annotated as “toxin B”. For VPI_9048, locus_tag H476_0269 [cytotoxin L (TcsL)] and H476_0271 [Hemorrhagic toxin (TcsH)] are annotated as toxin B [52]. In the strain *C. sordellii* 8483 locus tags WS9_01807 to WS9_01812 corresponds to toxin B and locus tag WS9_01787 correspond to toxin A. However, *C. sordellii* being a draft genomes, the locus tags in *C. sordellii* strains VPI_9048 and 8483 represent partial sequence of the toxin genes.

Four protein families homologous to collagenase (*colA*), sialidase (*nanH*), perfringolysin O (*pfoA*), and phospholipase C (*plc*), respectively, from *C. perfringens* were present in *C. sordellii* but absent in *C. difficile*. At the genome level, *C. sordellii* contains genes encoding enzymes for host tissue lysis and nutrient release during infection (e.g., hyaluronidase and hemolysin) [61]. The presence of these enzymes coupled with the ability to metabolize a larger set of peptides is likely to be a contributing factor in the ability of *C. sordellii* to cause lethal soft tissue infections. In addition, a cluster of eight genes encoding urease subunits (UreA (λ), UreB (β) and UreC (α)) and urease accessory proteins (UreI, UreE, UreF, UreG and UreH) homologous to known virulence factors of *Helicobacter pylori* 26695 (Enzyme; Acid resistance; Colonization) were present in *C. sordellii* but absent in *C. difficile*.

#### Clusters of Orthologous Groups (COG)

The Clusters of Orthologous Groups (COG) database [62] defines four major functional categories: “information storage and processing”, “cellular processes and signaling”, “metabolism”, and “poorly characterized”, which are further subdivided into 25 functional categories. Of the 4368 protein families, 1970 were assigned to the COG functional categories (Additional file 4: Table S4). The 211 protein families were assigned to COG functional category E (Amino acid transport and metabolism), of which 36 protein families were present in *C. sordellii* but absent in *C. difficile* (Table 1). *C. sordellii* can better metabolize dipeptides than *C. difficile* as illustrated in Fig. 2. Several *C. sordellii* protein families including ABC-type transport systems for dipeptides or amino acid, a variety of peptidases, and amino acid degradation enzymes could be responsible. A *C. sordellii* L-serine deaminase might facilitate not only improved utilization of serine as a nitrogen source but also improved glycine and threonine metabolism within the di-peptide analysis. A branched chain amino acid aminotransferase from *C. sordellii* might explain the improved metabolism of isoleucine and valine. The abundance in amino acid associated functions are consistent with our finding in the phenotype level that *C. sordellii* has more capacity to use a wider range of amino acids and peptides as nutrient sources than *C. difficile*.

**Table 1:** Protein families assigned to COG functional category E (Amino acid transport and metabolism) that are present in C. sordellii strain 8483 but absent in C. difficile strain 630

COG functional annotation
COG0076E|Glutamate decarboxylase and related PLP-dependent proteins
COG0115EH|Branched-chain amino acid aminotransferase/4-amino-4-deoxychorismate lyase
COG0346E|Lactoylglutathione lyase and related lyases
COG0477GEPR|Permeases of the major facilitator superfamily
COG0493ER|NADPH-dependent glutamate synthase beta chain and related oxidoreductases
COG0549E|Carbamate kinase
COG0697GER|Permeases of the drug/metabolite transporter (DMT) superfamily
COG0703E|Shikimate kinase
COG0747E|ABC-type dipeptide transport system, periplasmic component
COG0757E|3-dehydroquinate dehydratase II
COG0804E|Urea amidohydrolase (urease) alpha subunit
COG0831E|Urea amidohydrolase (urease) gamma subunit
COG0832E|Urea amidohydrolase (urease) beta subunit
COG0834ET|ABC-type amino acid transport/signal transduction systems, periplasmic component/domain
COG1104E|Cysteine sulfinate desulfinase/cysteine desulfurase and related enzymes
COG1410E|Methionine synthase I, cobalamin-binding domain
COG1703E|Putative periplasmic protein kinase ArgK and related GTPases of G3E family
COG1760E|L-serine deaminase
COG2235E|Arginine deiminase
COG2755E|Lysophospholipase L1 and related esterases
COG2856E|Predicted Zn peptidase
COG2866E|Predicted carboxypeptidase
COG2986E|Histidine ammonia-lyase
COG2987E|Urocanate hydratase
COG3033E|Tryptophanase
COG3191EQ|L-aminopeptidase/D-esterase
COG3227E|Zinc metalloprotease (elastase)
COG3643E|Glutamate formiminotransferase
COG4401E|Chorismate mutase
COG4448E|L-asparaginase II
COG4608E|ABC-type oligopeptide transport system, ATPase component
COG0141E|Histidinol dehydrogenase

### Urease gene cluster in environmental adaptation

**Fig. 8:**
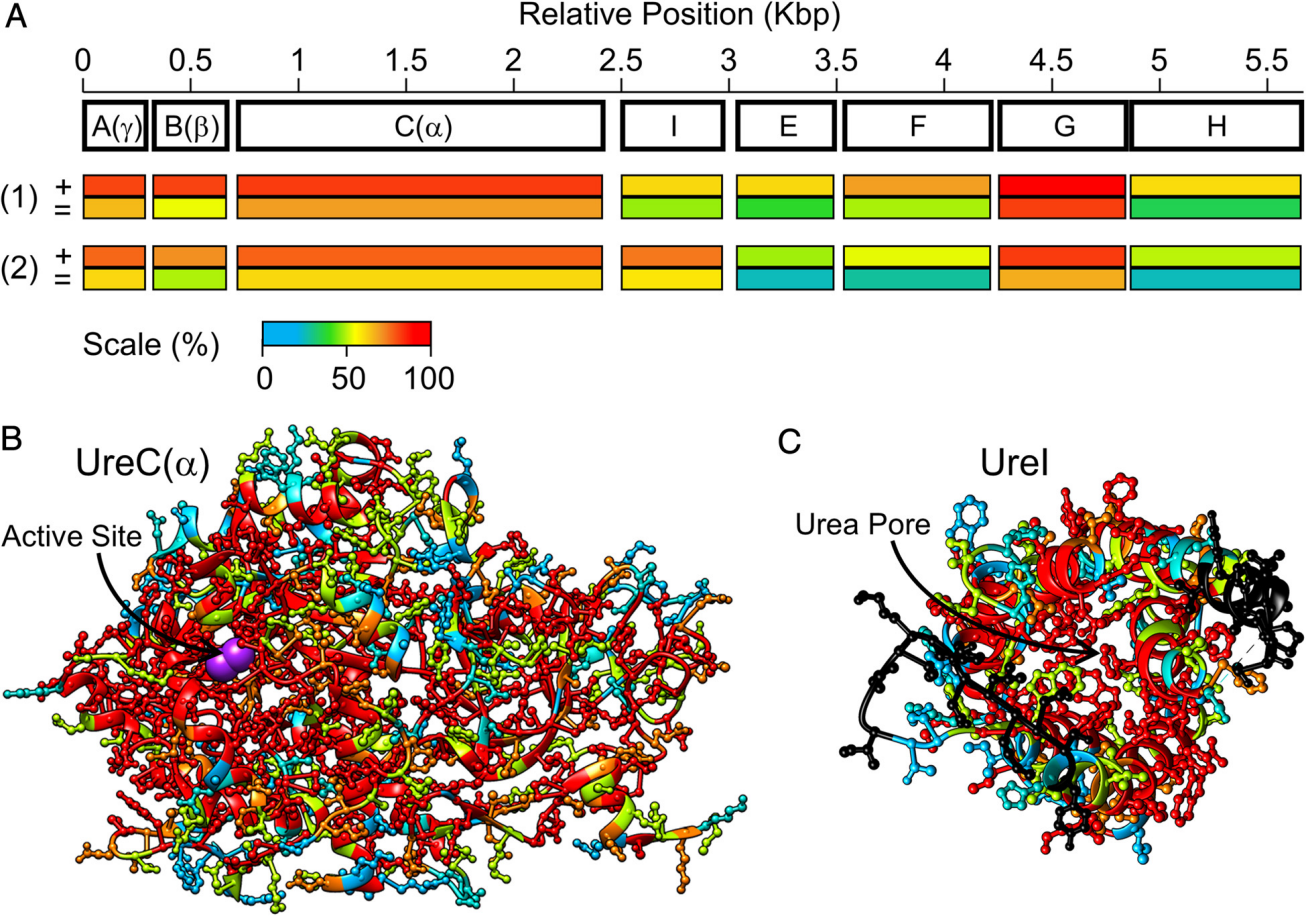
Urease gene cluster. **a** The size and organization of *C. sordellii* urease genes is illustrated. Overall homology of individual proteins to (1) *H. pylori* and (2) *C. perfringens* is depicted by colored bars for (+) % similarity and (=) % identity located below each gene. The scale is defined with red > 90 % and cyan < 20 %. **b** The urease enzyme structure from *H. pylori* (PDBID: 1E9Z) [66] and the urea channel structure from *H. pylori* (PDBID: 3UX4) [67] are both shown to illustrate the degree of conservation with corresponding *C. sordellii* urease proteins near the functional sites. Using the same scale for **a**, residues colored in red are conserved across *H. pylori*, *C. perfringens* and *C. sordellii*, while residues colored in cyan are not conserved

**Fig. 9:**
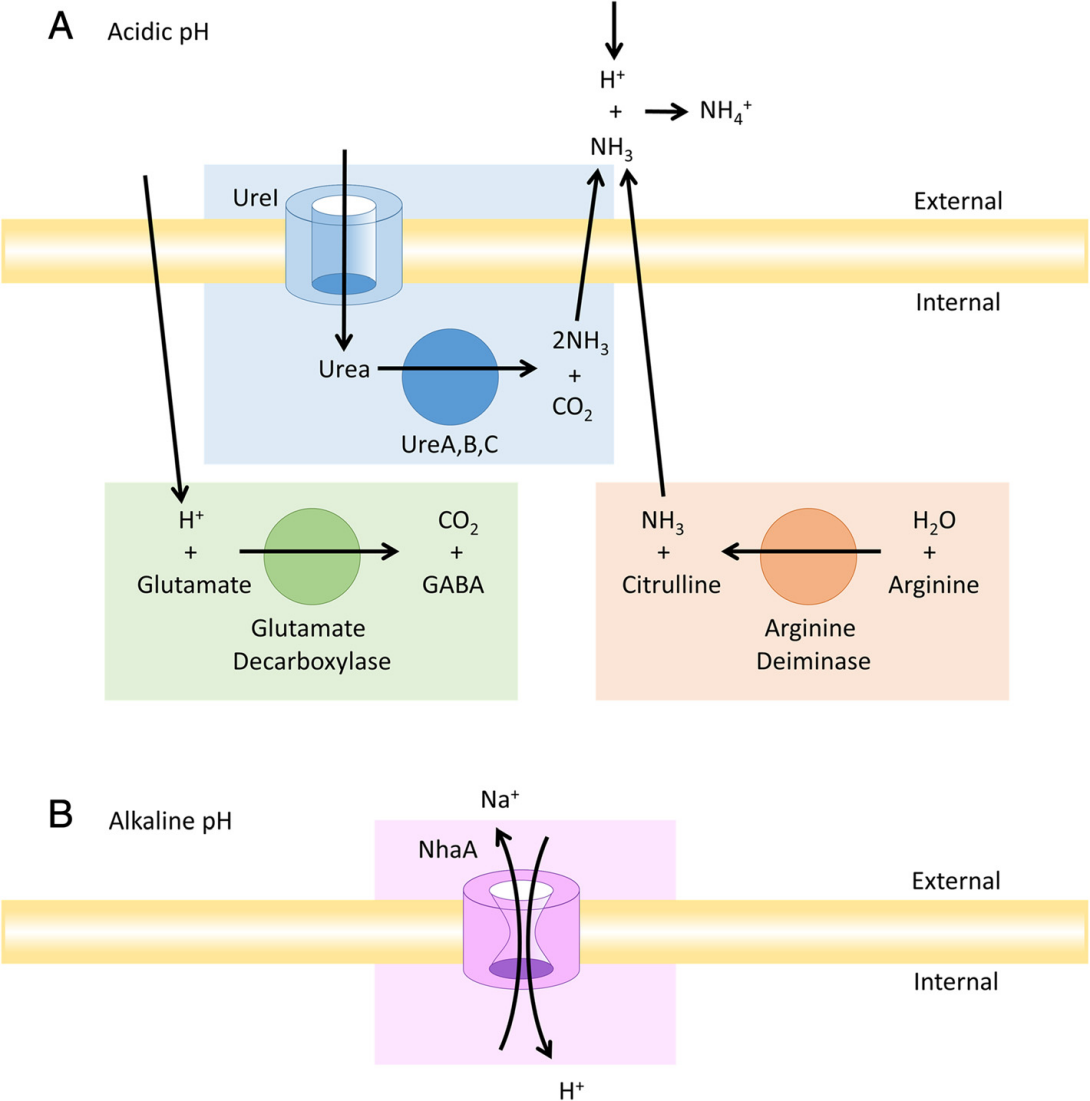
Possible mechanisms of acid adaptation. Several *C. sordellii* genes could be utilized to sequester protons and improve the internal and surrounding pH. **a** The expected contributions of urease, glutamate decarboxylase, and arginine deiminase to increasing the cytoplasmic pH under acidic conditions are depicted. **b** The NhaA Na^+^/H^+^ antiporter is expected to improve alkaline adaptation

Historically, a high level of urease activity has been used in the positive taxonomic identification of *C. sordellii*, although a number of urease-negative *C. sordellii* isolates have been identified [43]. A cluster of eight genes in *C. sordellii* strain 8483 was found to encode urease subunits: UreA (λ), UreB (β), UreC (α), UreI, UreE, UreF, UreG, and UreH (Fig. 8a). The organization of the urease gene cluster is similar to that of *Helicobacter* sp. [63, 64], yet the entire gene cluster was absent in *C. difficile*. Homologues to the eight urease genes are also present in *C. perfringens*; however, several of these urease genes are positioned on a large plasmid [65]. The set of urease proteins play an important function in acid resistance while also contributing a significant nitrogen source [53, 63]. UreA (λ), UreB (β), and UreC (α) form the core urease complex involved in conversion of urea to ammonia and carbonic acid [66]. Cytoplasmic urease activity substantially increases the intracellular pH and is essential for survival of many acidophilic pathogens including *Helicobacter pylori*. The proteins in the *C. sordellii* urease complex are highly homologous to *C. perfringens* and *H. pylori* (Fig. 8a) and share the highest degree of conservation near the enzyme active site (Fig. 8b). UreI forms a urea channel that allows efficient entrance of urea into the cytoplasm for urease degradation [67]. UreI is also homologous to *C. perfringens* and *H. pylori* with a high degree of conservation for residues lining the pore (Fig. 8c). The relatedness of proteins from the *C. sordellii* urease gene cluster to other pathogen urease proteins (particularly near functional residues) suggests a similar role in low pH tolerance (Fig. 9). In addition, urea is a byproduct of protein degradation creating a potential nitrogen source during infection. The two major phenotypes that provide *C. sordellii* the opportunity to colonize the vagina and soft tissue are the ability to survive below pH 4.5 and to utilize peptides as nitrogen sources. These two functions likely make the urease gene cluster important in establishing and maintaining *C. sordellii* infections. Future studies directed at disrupting the *C. sordellii* urease gene cluster (i.e., gene knockouts) should better define the gene cluster’s role in the low pH tolerance exhibited by *C. sordellii*.

### Other genes involved in environmental stress tolerance

Several other *C. sordellii* genes that are absent from *C. difficile* could play a role in adaptation to acidic conditions (Table 1; Fig. 9a). Glutamate decarboxylase converts glutamate to γ-aminobutyric acid (GABA) which absorbs a proton during the reaction. Arginine deiminase generates ammonia which can also absorb a proton and create a more alkaline internal, periplasmic, or local pH in an acidic environment. Some bacteria can accumulate high cytoplasmic potassium levels under acidic stress. Potassium transport is expected to play a role in adaptation to acidic environments by maintaining the membrane potential for optimum bioenergetics homeostasis [68, 69], yet the exact mechanism of how the various transporters and channels work together to support the internal pH is not fully understood. Potassium homeostasis is also pivotal in the osmotic stress response.

Similar growth of *C. sordellii* strain 8483 from pH 6 to a more alkaline pH range was observed (Fig. 3). A homologue of the Na^+^/H^+^ antiporter, NhaA, was identified in the genome of *C. sordellii* strains 8483, ATCC 9714 and VPI 9048 but was identified only in *C. difficile* strain F501. In *Escherichia coli* and *Salmonella enterica*, NhaA has been implicated as a mechanism for maintaining internal pH homeostasis under alkaline conditions by catalyzing H^+^ uptake for a preferred pH_out_ range of 6.5 to 8.5 [70, 71]. The presence of NhaA in all *C. sordellii* strains but absence from most *C. difficile* strains could explain the ability of *C. sordellii* to maintain growth levels at high pH (Fig. 9b). Finally, some of the observed phenotypic differences could be multifactorial and related to differences in the gene expression levels of many genes. As is the case with the activation of toxin genes in *C. difficile*, gene expression levels are likely to be correlated to several components in the bacterium's nutritional environment, such as the presence of sugars, amino acids, and fatty acids [72–74].

## Conclusions

The related pathogens, *C. difficile* and *C. sordellii*, were compared through the analysis of phenomic and genomic datasets. While *C. difficile* infections have been well studied, significantly less information regarding *C. sordellii* infections is available. In particular, the current study focused on uncovering the basis for *C. sordellii*‘s preference for infecting both soft tissues and the vagina, while not infecting the gastrointestinal tract (a major clinical difference between *C. difficile* and *C. sordellii*). A comparison of the phenome between *C. difficile* and *C. sordellii* revealed that *C. sordellii* had adapted to survive under conditions that require the procurement of resources from host tissue. In addition, *C. sordellii* can withstand more acidic pH than *C. difficile* thereby allowing it to survive in the low pH environment of the vagina. The complementary genomic analysis revealed a large number of proteins present in *C. sordellii* but not in *C. difficile* that are likely to play an adaptive role in metabolism and pH tolerance. In this context, the urease gene cluster is described in detail. The phenomic and genomic comparison between *C. difficile* and *C. sordellii* should provide guidance for the development of targeted treatments for Clostridial infections.

## Methods

### Bacterial culturing and phenotype microarray experiments

The global nutritional phenome of *C. sordellii* strain 8483 was measured using Biolog Phenotype microarrays (PMs). *C. sordellii* strain 8483 is a human blood isolate obtained from the United Sates Centers for Disease Control and Prevention. PM Technology consists of different PM panels, of which PMs 1–8 are linked to nutrient utilization (metabolism) and PMs 9–10 are related to chemical sensitivity. We have used PMs 1–8 to analyze the global nutritional phenome of *C. sordellii* and *C. difficile* and PMs 9–10 to test osmolyte and pH sensitivities. All experiments were conducted in a Bactron IV anaerobic chamber (Shell Lab, OR). Prior to PM experiments, *C. sordellii* 8483 was grown in anaerobic Brain Heart Infusion (BHI) broth. PM experiments were performed following standard Biolog Inc. protocol [35]. Briefly, 300 μl of the bacteria grown in BHI broth was plated on Biolog Universal blood agar plates and was incubated overnight at 37 °C. A 40 % transmittance cell suspension in Biolog solution IF-0a was then prepared by re-suspending bacteria grown on Biolog Universal blood agar plates. This suspension was then diluted with Biolog mix B at a ratio of 1:16 and then transferred to each of the 96-well PM microplates (a set of 95 substrates and one blank well). The inoculation volume was 100 μl/well. The plates were incubated at 37 °C for 48 h. The optical density (OD) values were then measured at 750 nm using an ELISA reader. Each experiment was performed as biological triplicates.

For statistical analysis of the PM data, means of the replicates were taken. For normalizing the data between strains, each PM well’s mean was divided with the mean of the respective plates negative control. The value of each well was then compared using ANOVA to the negative control value of the respective plate. A PM well was considered positive if its value was 40 % higher than the negative control at 5.0 % significance level. The Model SEED database [75] was then used to predict the genome scale metabolic phenotype of *C. sordellii* and *C. difficile*. For phenotype comparisons of *C. difficile* with *C. sordellii*, sum of positive phenotypes of *C. difficile* strains [38] was taken. We compared the predicted metabolic phenotypes with the positive PM results. This comparison showed that at 40 % growth increase cut off from negative control, false positives are completely avoided.

### Genomic DNA isolation, genome sequencing and data collation

For isolating genomic DNA, *C. sordellii* strain 8483 was streaked on BHI agar plate and incubated anaerobically at 37 °C overnight. A single colony from this plate was then used to inoculate BHI broth and was incubated at anaerobic conditions for 12 h. From 1.0 ml of this culture, following manufacturer’s protocol, genomic DNA was isolated using MasterPure™ Gram Positive DNA Purification Kit (Epicenter Biotechnologies, Madison, WI). Roche/454 pyrosequencing, involving paired-end reads from the FLX sequencer, was used to determine the genome sequence of *C. sordellii* strain 8483 with sequencing coverage of 35x. The sequences were assembled De novo using Newbler Software Release: 2.5.3. Genome annotation for the strain was done by the National Center for Biotechnology Information (NCBI) Prokaryotic Genomes Automatic Annotation Pipeline. The *C. sordellii* whole genome shotgun project has been deposited at DDBJ/EMBL/GenBank under the accession AJXR00000000. The version described in this paper is the first version, AJXR01000000.

We used Bioperl version 1.6.1 [76] and G-language Genome Analysis Environment version 1.8.13 (http://www.g-language.org) [77–79] for sequence data analysis, and R version 3.1.0 for statistical computing (http://www.R-project.org) [80]. For comparative analysis, bacterial genome sequences in GenBank format [81] were retrieved from the NCBI FTP site (ftp://ftp.ncbi.nih.gov/) and from the PATRIC [82] FTP site (ftp://ftp.patricbrc.org/patric2/genomes/). Protein-coding sequences were retrieved from the bacterial genomes. Homologous proteins were identified by the BLAST (Basic Local Alignment Search Tool) program [83] with an E value cutoff of 1e-5 and a minimum aligned sequence length coverage of 50 % of a query sequence.

### Phylogenetic analysis

A group of orthologous proteins was built by all-against-all protein sequence comparison using BLASTP followed by FastOrtho with default parameters (http://enews.patricbrc.org/fastortho/), which is a reimplementation of the OrthoMCL program [84]. We used the 351 ortholog groups shared by all the strains and contained only a single copy from each strain. These orthologs were aligned as follows: i.e., nucleotide sequences are translated into amino acid sequences, aligned with MUSCLE [85, 86], back translated into nucleotide sequences, and ambiguous regions (containing gaps and poorly aligned) were eliminated with Gblocks [87, 88]. The orthologs with more than 50 % of their regions removed are disregarded from the phylogenetic analysis. This retained 346 reliably aligned orthologs from a set of the 351 orthologs. A phylogenetic tree for each of the 346 orthologous genes (gene tree) was reconstructed using RAxML [89] with the GTRGAMMA model. A majority-rule consensus (extended) of the gene trees was constructed using consense program of PHYLIP 3.69 [90]. Because the selection of genes with stronger phylogenetic signal reduced incongruence [91], we analyzed the data set of comprising genes whose bootstrap consensus trees showed average bootstrap support across all internodes that was greater than or equal to 90 % (134 genes). The alignments from the set of the 134 orthologous genes were concatenated, and a tree search was performed using RAxML with the same settings as for the individual gene trees. Phylogenetic trees were drawn using DendroPy [92] and the R package APE (Analysis of Phylogenetics and Evolution) [93].

### Gene repertoire analysis

A group of homologous proteins (protein family) was built by all-against-all protein sequence comparison using BLASTP followed by Markov clustering (MCL) [94] with an inflation factor of 1.2 using MCLBLASTLINE (http://micans.org/mcl/). To detect missed protein-coding sequences due to differences in gene finding algorithms [95], we performed TBLASTN homology searches of each strain’s proteins against the other strain’s whole nucleotide sequence. The resulting gene content (binary data, 1 or 0, representing presence or absence of each protein family) is shown in Additional file 4: Table S4.

We used Jaccard distance (one minus Jaccard coefficient) to measure a distance between two genomes based on binary data, 1 or 0, representing the presence or absence of each protein family for each genome (gene content). The resulting distance matrix was subject to a neighbour-joining clustering and hierarchical clustering with three agglomeration methods (i.e., single-, complete-, and average-linkage clustering), and dendrograms were drawn to visualize the clustering results.

### Gene functional annotation

We assigned functional annotations to each protein family by merging all the functional annotations of proteins belonging to the same family. To gain different aspects and maximize coverage, protein families were annotated by multiple databases. We performed BLASTP searches of protein sequences against NCBI nr (non-redundant) database, COG [62], KEGG [39], UniProtKB/Uniref90 [96], Virulence Factors Database (VFDB) [60], and assigned the functional annotations of the most similar protein sequences in each database. We converted protein_ID to subsystems (Category, Subcategory, Subsystem, and Role) in SEED database [97]. We also searched protein sequences against the Pfam library of hidden Markov models (HMMs) [98] using HMMER, and mapped Gene Ontology (GO) terms to Pfam entries using the ‘pfam2go’ mapping provided by the GO consortium [99].

### Availability of supporting Data

The data sets supporting the results of this article are included within the article and its additional files. The genome sequence data for the *C. sordellii* whole genome shotgun project has been deposited at DDBJ/EMBL/GenBank under the accession AJXR00000000. The version described in this paper is the first version, AJXR01000000. The phylogenetic trees described in this manuscript have been deposited to TreeBase. Access to the data is available upon publication at http://purl.org/phylo/treebase/phylows/study/TB2:S17636.

## Additional files

Additional file 1: Table S1. Comparison of nutritional, osmolyte, and pH phenotype values tested using Biolog Phenotype microarrays (PMs) for *C. sordellii* strain 8483 and *C. difficile* strain 630. Phenotypes are colored green (positive) and red (negative). Additional file 2: Table S2. Positive counts for the 854 nutritional phenotypes tested using Biolog Phenotype microarrays (PMs) for *C. sordellii* strain 8483 and *C. difficile* strain 630. Additional file 3: Table S3. Genomic features for bacterial species analyzed. Additional file 4: Table S4. Gene content table for eight *C. difficile* strains (630, BI1, CD196, M68, R20291, 2007855, CF5, and M120) and three *C. sordellii* strains (8483, ATCC_9714, and VPI_9048). The first 11 columns contain the protein family identification number (Family No.), locus_tag, amino acid length (Laa), functional annotations from different databases (GenBank, COG, KEGG, SEED, VFDB, Pfam, GO, and UniProt). The remaining columns show binary data (1 or 0) for presence or absence of each protein family for each strain. Additional file 5: Table S5. Database categories that are over- or underrepresented in *C. sordellii* 8483 relative to *C. difficile* 630. a = the number of *C. sordellii* protein families in this category, b = the number of *C. sordellii* protein families not in this category, c = the number of *C. difficile* protein families in this category, d = the number of *C. difficile* protein families not in this category, odds ratio = ad/bc, P-value obtained by Fisher’s exact test, and false discovery rate (FDR) adjusted p-value.

## Abbreviations

BHI: Brain Heart Infusion
colA: collagenase
CDI: *C. difficile* infectio
CSI: *C. sordellii* infection
GO: Gene Ontology
GABA: γ-aminobutyric acid
HMMs: hidden Markov models
MCL: Markov clustering
*pfo*A: perfringolysin O
PMs: Phenotype microarrays
*plc*: phospholipase C
CDS: protein-coding sequences
*nanH*: sialidase
VFDB: Virulence Factors Database
